# Correction: Luedemann et al. Prostate Cancer-Associated miRNAs in Saliva: First Steps to an Easily Accessible and Reliable Screening Tool. *Biomolecules* 2022, *12*, 1366

**DOI:** 10.3390/biom13040628

**Published:** 2023-03-31

**Authors:** Christoph Luedemann, Jan-Ludwig Reinersmann, Claudia Klinger, Stephan Degener, Nici Markus Dreger, Stephan Roth, Michael Kaufmann, Andreas Savelsbergh

**Affiliations:** 1 Department of Internal Medicine, Gastroenterology, Hepatology, Infectiology, Nephrology, Endocrinology & Diabetology, University Hospital of Bonn, 53127 Bonn, Germany; 2 Division of Functional Genomics, Chair for Biochemistry and Molecular Medicine, University of Witten/Herdecke, 58453 Witten, Germany; 3 Department of General, Visceral and Vascular Surgery, Marienhospital Brühl, 50321 Brühl, Germany; 4 Center for Biochemical Education and Research (ZBAF), University of Witten/Herdecke, 58453 Witten, Germany; 5 Department of Urology, Helios University Hospital Wuppertal, University of Witten/Herdecke, 42283 Wuppertal, Germany

The authors wish to make the following corrections to their paper [1]. Eight sequences were missed in the original Table 3. The correct version appears below. The authors state that the scientific conclusions are unaffected. This correction was approved by the Academic Editor. The original publication has also been updated.

## Figures and Tables

**Table 3:** Primer assays ID and sequence, acquired from Qiagen^®^.

microRNA	Mature	Accession	Sequence
MIR106A	hsa-miR-106a-5p	MIMAT0000103	13-AAAAGUGCUUACAGUGCAGGUAG-35
MIR130B	hsa-miR-130b-5p	MIMAT0004680	13-ACUCUUUCCCUGUUGCACUAC-33
MIR301A	hsa-miR-301a-5p	MIMAT0022696	14-GCUCUGACUUUAUUGCACUACU-35
MIR331	hsa-miR-331	MIMAT0000760	61-GCCCCUGGGCCUAUCCUAGAA-81
MIR326	hsa-miR-326	MIMAT0000756	60-CCUCUGGGCCCUUCCUCCAG-79
MIR375	hsa-miR-375-3p	MIMAT0000728	40-UUUGUUCGUUCGGCUCGCGUGA-61
MIR484	hsa-miR-484	MIMAT0002174	8-UCAGGCUCAGUCCCCUCCCGAU-29
MIR2110	hsa-miR-2110	MIMAT0010133	8-UUGGGGAAACGGCCGCUGAGUG-29
MIR107	hsa-mir-107	MIMAT0000104	50-AGCAGCAUUGUACAGGGCUAUCA-72
MIR622	hsa-mir-622	MIMAT0003291	61-ACAGUCUGCUGAGGUUGGAGG-81
MIR141	hsa-mir-141	MIMAT0000432	5ß-UAACACUGUCUGGUAAAGAUGG-38
MIR432	hsa-mir-432	MIMAT0002814	14-UCUUGGAGUAGGUCAUUGGGUGG-36
MIR574	hsa-mir-574-3p	MIMAT0003239	61-CACGCUCAUGCACACACCCACA-82
MIR625	hsa-mir-625	MIMAT0003294	15-AGGGGGAAAGUUCUAUAGUCC-35
MIR181A	hsa-mir-181a-2-3p	MIMAT0004558	77-ACCACUGACCGUUGACUGUACC-98
MIR200b	hsa-mir-200b	MIMAT0000318	57-UAAUACUGCCUGGUAAUGAUGA-78
